# Supplementary material for: Development and Validation of a Knowledge, Attitude and Practice Questionnaire on Antibiotic Use in Arabic and French Languages in Lebanon
Source: Int J Environ Res Public Health. 2022 Jan 8;19(2):687. doi: 10.3390/ijerph19020687 (PMC8776152; doi:10.3390/ijerph19020687)
Supplement: Supplementary file 1 [file ijerph-19-00687-s001.zip › File S1.pdf]

CONNAISSANCES, CROYANCES ET UTILISATION D'ANTIBIOTIQUES AU LIBAN

Code du questionnaire:_________________ Date:__________________________

Pour choisir une réponse, veuillez remplir <u>COMPLÈTEMENT</u> le(s) cercle(s) correspondant(s): CORRECTEMENT répondu: ●
INCORRECTEMENT répondu: ⊗ ∅

Veuillez répondre aux questions suivantes sur les *ANTIBIOTIQUES* (Amoxicillin, Cefixime, Ciproflaxin, Levoflaxin, Penicillin, etc…)

Sur une échelle de 0 à 10 points, sélectionnez un chiffre représentant votre niveau d'accord avec les affirmations ci-dessous. "0" (zéro) veut dire que vous n'êtes pas du tout d'accord et "10" représente un accord maximum.

Veuillez indiquer votre niveau d'accord **<u>QUE VOUS</u>** utilisiez des *antibiotiques* **<u>OU PAS</u>**

Exemple: Faire du sport est bénéfique pour la santé ⓪ ① ② ③ ④ ⑤ ⑥ ⑦ ● ⑨ ⑩

		Pas d'accord ←										→ D'accord
		0	1	2	3	4	5	6	7	8	9	10
**1.**	**Les *antibiotiques* sont efficaces contre les virus**	○	○	○	○	○	○	○	○	○	○	○
**2.**	**Quand je suis enrhumé(e) ou j'ai une grippe, je prends des *antibiotiques* pour m'aider à me sentir mieux plus vite**	○	○	○	○	○	○	○	○	○	○	○
**3.**	**Si je me sens mieux après quelques jours de prise d'*antibiotiques*, il m'arrive d'arrêter mon traitement avant de le finir**	○	○	○	○	○	○	○	○	○	○	○
**4.**	**J'attends de mon médecin qu'il me prescrive des *antibiotiques* quand j'ai des symptômes de rhume ou une grippe**	○	○	○	○	○	○	○	○	○	○	○
**5.**	**Il est bon de pouvoir obtenir des *antibiotiques* de parents ou amis sans devoir voir un médecin**	○	○	○	○	○	○	○	○	○	○	○
**6.**	**Quand j'ai un mal de gorge, je préfère prendre un *antibiotique***	○	○	○	○	○	○	○	○	○	○	○
**7.**	**Chaque type d'infection a besoin d'un *antibiotique* différent**	○	○	○	○	○	○	○	○	○	○	○
**8.**	**Les *antibiotiques* peuvent tuer les bactéries qui vivent habituellement sur la peau et dans les intestins**	○	○	○	○	○	○	○	○	○	○	○
**9.**	**Si je ressens des effets secondaires durant un traitement d'antibiotiques, je dois arrêter de prendre le médicament dès que possible**	○	○	○	○	○	○	○	○	○	○	○
**10.**	**Je prends les *antibiotiques* en suivant les instructions du médecin**	○	○	○	○	○	○	○	○	○	○	○
**11.**	**Quand les *antibiotiques* sont consommés de façon excessive, ils n'auront plus d'effet au moment où l'on en a vraiment besoin**	○	○	○	○	○	○	○	○	○	○	○
**12.**	**Je préfère garder des *antibiotiques* chez moi au cas où j'en aurais besoin plus tard**	○	○	○	○	○	○	○	○	○	○	○
**13.**	**Je fais confiance au médecin s'il /si elle décide de prescrire ou pas des *antibiotiques***	○	○	○	○	○	○	○	○	○	○	○
**14.**	**Si je pense que j'ai besoin d'un *antibiotique* et que le médecin ne me le prescrit pas, je vais à la pharmacie pour me le procurer sans ordonnance**	○	○	○	○	○	○	○	○	○	○	○
**15.**	**Les médecins expliquent souvent clairement au patient les raisons de prescrire ou de ne pas prescrire des antibiotiques**	○	○	○	○	○	○	○	○	○	○	○
**16.**	**Les médecins expliquent souvent clairement au patient les instructions d'utilisation des *antibiotiques***	○	○	○	○	○	○	○	○	○	○	○
**17.**	**Quand le patient achète un *antibiotique*, le pharmacien lui explique l'importance d'une bonne adhésion thérapeutique**	○	○	○	○	○	○	○	○	○	○	○

18. Au cours du mois dernier, avez-vous pris des *antibiotiques*?

○ Oui (passer à la question 19) ○ Non (passer à la question 29)

Veuillez répondre aux questions suivantes sur votre consommation des *ANTIBIOTIQUES* AU COURS DU MOIS DERNIER

19. Quelle a été la durée de votre dernier traitement aux *antibiotiques*?

Nombre de jours ☐☐ Nombre de mois ☐☐

20. Qui vous a prescrit ou recommandé les *antibiotiques*? (Vous pouvez choisir plusieurs réponses)

○ Le médecin ○ Des amis
○ La famille ○ Le pharmacien
○ Je les avais chez moi

21. Avez-vous pris jusqu'au bout votre dernier traitement aux *antibiotiques*?

○ Oui ○ Non

22. Qu'avez-vous fait des *antibiotiques* que vous n'avez pas utilisés? (Vous pouvez choisir plusieurs réponses)

○ Je les ai gardés pour les utiliser la prochaine fois que je suis malade
○ Je m'en suis débarrassé(e) dans la poubelle ou les eaux usées
○ Je les ai donnés à quelqu'un d'autre
○ Il ne me restait plus d'antibiotique

23. Au cours de votre dernier traitement aux *antibiotiques*, vous est-il arrivé d'oublier de les prendre?

○ Jamais (passer à la question 25)
○ Parfois (passer à la question 24)
○ Souvent (passer à la question 24)

24. Qu'avez-vous fait quand vous avez oublié de prendre une dose de vos *antibiotiques*?

○ J'ai continué de prendre les doses suivantes normalement
○ J'ai doublé/augmenté la dose suivante
○ Je l'ai prise dès que je m'en suis souvenu(e)

25. Au cours de votre dernier traitement aux antibiotiques, avez-vous changé la dose sur votre propre décision (sans avis médical)**?**

○ Jamais (passer à la question 29)
○ Parfois (passer à la question 26)
○ Souvent (passer à la question 26)

26. Comment avez-vous vous-même changé votre dose d'*antibiotiques* (sans avis médical)**?**

○ J'en ai pris plus (passer à la question 27 puis 29)
○ J'en ai pris moins (passer à la question 28)
○ Parfois j'en ai pris plus et parfois moins (passer à la question 27 puis 28)

27. Pourquoi avez-vous vous-même décidé d'augmenter votre dose d'*antibiotiques* (sans avis médical)**?** (vous pouvez choisir plusieurs réponses)

○ J'ai oublié de prendre la dose précédente
○ Je me suis senti(e) très malade
○ Je n'ai pas senti de réelle amélioration
○ J'ai senti une amélioration mais voulais me sentir encore mieux

28. Pourquoi avez-vous décidé vous-même de réduire votre dose d'*antibiotiques* (sans avis médical)**?** (vous pouvez choisir plusieurs réponses)

○ J'avais peur des effets secondaires
○ J'étais fatigué(e) et j'ai oublié
○ J'ai pensé que j'en prenais trop
○ J'étais pressé(e) et j'ai oublié
○ Je n'aime pas prendre de médicaments la nuit
○ Je me sentais mieux

Caractéristiques Démographiques Générales

29. Indiquez votre sexe ○ Homme ○ Femme

30. Quelle est votre date de naissance? (Veuillez inscrire les chiffres dans les espaces alloués)

□□	□□	□□□□
**Jour**	**Mois**	**Année**

OMɸ Quel est votre niveau d'éducation académique.

- ○ Je n'ai pas été à l'école
- ○ Elémentaire/ Primaire (maternelle jusqu'à la 6ème)
- ○ Complémentaire (5ème à la 3ème)
- ○ Secondaire (2nde à la terminale)
- ○ Universitaire

OHɸ Actuellement, avez-vous une activité professionnelle?

○ Oui ○ Non

OOɸ Qu'est-ce qui est couvert par votre assurance santé? (Vous pouvez choisir plus d'une réponse).

- ○ Je n'ai pas d'assurance santé
- ○ Le coût ou une partie des coûts des consultations médicales
- ○ Le coût ou une partie des coûts de mes médicaments

34. Veuillez indiquer votre statut marital:

- ○ Marié(e) (passer à la question 35)
- ○ Divorcé(e) (passer à la question 37)
- ○ Veuf/Veuve (passer à la question 37)
- ○ Séparé(e) de mon épouse/époux, mais non divorcé(e) (passer à la question 35)

35ɸ Quel est le niveau d'éducation académique de votre époux/épouse?

- ○ Il/Elle n'a pas été à l'école
- ○ Elémentaire/ Primaire (maternelle jusqu'à la 6ème)
- ○ Complémentaire (5ème à la 3ème)
- ○ Secondaire (2nde à la terminale)
- ○ Universitaire

06ɸ Actuellement, votre époux/épouse travaille-t-il/elle?

○ Oui ○ Non

37ɸ Combien de personnes vivent sous votre toit, inclus vous-même?

○ 2 ○ 3 – 4 ○ 5 – 6 ○ Plus de 6

38. Y a-t-il des instituts de santé, tels que des dispensaires ou hôpitaux, situés près de votre lieu de résidence?

○ Oui ○ Non

39. Quel est le total des revenus familiaux (quel est le revenu total perçu par tous les membres de la famille vivant sous le même toit)?

- ○ Moins de 500US$ par mois
- ○ Entre 500 et 999US$ par mois
- ○ Entre 1000 et 1499US$ par mois
- ○ Entre 1500 et 1999US$ par mois
- ○ Entre 2000 et 2500US$ par mois
- ○ Plus de 2500US$ par mois

40. Consultez-vous un médecin lorsque vous êtes malade?

○ Jamais ○ Rarement

○ Parfois ○ Toujours (passer à la question 42)

41. Quelle est la raison pour laquelle vous ne consultez pas toujours un médecin? (Vous pouvez choisir plus d'une réponse).

- ○ Je n'ai pas besoin de médecin
- ○ J'ai peur des médecins
- ○ Par manque d´argent
- ○ Je n'ai pas le temps de le consulter
- ○ L'attente avant d'être examiné(e) est longue
- ○ La clinique est située loin de ma maison

42. Avez-vous déjà reçu une prescription médicale par téléphone?

○ Oui ○ Non

Merci d'avoir participé à cette étude !
