# Supplementary material for: Development and Validation of a Knowledge, Attitude and Practice Questionnaire on Antibiotic Use in Arabic and French Languages in Lebanon
Source: Int J Environ Res Public Health. 2022 Jan 8;19(2):687. doi: 10.3390/ijerph19020687 (PMC8776152; doi:10.3390/ijerph19020687)
Supplement: Supplementary file 1 [file ijerph-19-00687-s001.zip › File S2.pdf]

المَعرفَة وَالإعتِقَاد وَإسْتِخدَام المُضَادَّات الحَيَويَّة في لبنان

رمز الإستبيان:__________ التَّاريخ: ______________

للإجابة على سؤال، رجاءً إملأ الدَّائرة المقابلة بالكامل: أجِيبَ بِشَكْلٍ صَحِيح : ● أجِيبَ بِشَكْلٍ خاطئ: ⊗ ∅

يُرْجَى الإِجَابَة عَلَى الأسئِلَة التَّالِيَة حَوْلَ *المُضَادَّات الحَيَوِيَّة* (مثلاً: أموكسيسيلين، سيفيكسيم، سيبروفلوكساسين، ليفوفلوكساسين، بنسلين، إلى آخره) ؟

على مقياس من "⓪" (صفر) إلى "⑩"(عشر) نقاط، إختر رقم يعبر عن درجة موافقتك على العبارات التَّالية، بحيث أن الرقم "⓪" (صفر) يعني إنّك ترفض كُليّاً والرقم "⑩" (عشرة) يعني إنّك توافق تماماً

يُرجى تقييم العبارات التَّالية **بصرف النَّظر** عمّا إذا كُنت تستخدم *المُضَادَّات الحَيَوِيَّة* **أم لا**

مثلاً: مُمارسة ا لرِّياضة تُفيد الصِّحَّة ⓪ ① ② ③ ④ ⑤ ⑥ ⑦ ● ⑨ ⑩

	لا أُوافِق ←									→ أُوافِق
	0	1	2	3	4	5	6	7	8	9	10
١) *المُضَادَّات الحَيَوِيَّة* فعَّالة ضِدَّ الفيروسات	⓪	①	②	③	④	⑤	⑥	⑦	⑧	⑨	⑩
٢) عندما أُصاب بنزلات البرد أو الإنفلونزا أتناول *المُضَادَّات الحَيَوِيَّة* لمساعدتي على التحسُّن بشكل أسرع	⓪	①	②	③	④	⑤	⑥	⑦	⑧	⑨	⑩
٣) إذا شعرت بالتحسُّن بعد بضعة أيَّام، أتوقف أحيانًا عن تناول *المُضَادَّات الحَيَوِيَّة* قبل إنتهاء فترة العلاج	⓪	①	②	③	④	⑤	⑥	⑦	⑧	⑨	⑩
٤) أتوقَّع أن يصف لي الطّبيب *المُضَادَّات الحَيَوِيَّة* إذا كنت أشعر بعوارض نزلات البرد أو الإنفلونزا	⓪	①	②	③	④	⑤	⑥	⑦	⑧	⑨	⑩
٥) مِن الجَيِّد أن تكون قادراً على الحصول على *المُضَادَّات الحَيَوِيَّة* من الأقارب أو الأصدقاء دون الحاجة إلى زيارة الطّبيب	⓪	①	②	③	④	⑤	⑥	⑦	⑧	⑨	⑩
٦) عِنْدَمَا أشعر بالْتِهَاب فِي الحَلْق، أَفْضَل إِسْتِخْدَامِ *مُضَادّ حَيَوِيّ*	⓪	①	②	③	④	⑤	⑥	⑦	⑧	⑨	⑩
٧) كل نوع من الإلتهاب يحتاج إلى نوع مختلف من *المُضَادَّات الحَيَوِيَّة*	⓪	①	②	③	④	⑤	⑥	⑦	⑧	⑨	⑩
٨) *المُضَادَّات الحَيَوِيَّة* تقتل البكتيريا التي تعيش عادةً على الجلد أو في المُصْرَان	⓪	①	②	③	④	⑤	⑥	⑦	⑧	⑨	⑩
٩) إذا شعرت بآثار جانبيّة خلال فترة العلاج *بالمُضَادَّات الحَيَوِيَّة* يجب أن أتوقف عن إستعماله في أقرب وقت ممكن	⓪	①	②	③	④	⑤	⑥	⑦	⑧	⑨	⑩
١٠) أستعمل *المُضَادَّات الحَيَوِيَّة* حسب تعليمات الطّبيب	⓪	①	②	③	④	⑤	⑥	⑦	⑧	⑨	⑩
١١) إذا تمّ إستهلاك *المُضَادَّات الحَيَوِيَّة* بكثرةٍ، فلن تنفع عند الحاجة إليها حقّاً	⓪	①	②	③	④	⑤	⑥	⑦	⑧	⑨	⑩
١٢) أُفضّل أن أُبقي *مُضَادَّات حَيَوِيَّة* إضافيّة في المنزل، في حال الحاجة إليها لاحقاً	⓪	①	②	③	④	⑤	⑥	⑦	⑧	⑨	⑩
١٣) أثِق بقرار الطّبيب سواء قرّر وصف أو عدم وصف *المُضَادَّات الحَيَوِيَّة*	⓪	①	②	③	④	⑤	⑥	⑦	⑧	⑨	⑩
١٤) إذا كنت أعتقد أنّني بحاجة إلى *المُضَادَّات الحَيَوِيَّة* ولم يصفها الطّبيب، سأحصل عليها من الصَّيْدَلِيَّة بدون وصفة	⓪	①	②	③	④	⑤	⑥	⑦	⑧	⑨	⑩
١٥) غَالِبًا مَا يَشرَح الأَطبَّاء بُوضوح للمريض أَسباب وصف أَوْ عدم وصف *المُضَادَّات الحَيَوِيَّة*	⓪	①	②	③	④	⑤	⑥	⑦	⑧	⑨	⑩
١٦) غَالبًا مَا يَشرح الأ طبّاء بِوضوح للمريض كيفية إستخدام *المضادَّات الحيويَّ ة*	⓪	①	②	③	④	⑤	⑥	⑦	⑧	⑨	⑩
١٧) عند شراء *المضادَّات الحيويَّة*، يبلغك الصَّيْدَلانيّ عن أهمية التَّقيُّد الصَّحيح بالعلاج	⓪	①	②	③	④	⑤	⑥	⑦	⑧	⑨	⑩

١٨) في الشّهر الماضي، هل إستعملت *المُضَادَّات الحَيَوِيَّة*؟ ○ نعم (رجاءً إنتقل إلى السُّؤال ١٩) ○ كلا (رجاءً إنتقل إلى االسُّؤال ٢٩)

أَجِب على الأَسْئِلَةِ التَّالِيَة عن إستهلاكك من المُضَادَّات الحَيَوِيَّة في الشّهر الماضي

١٩) كم كانت مُدَّة آخِرعلاج *بالمُضَادَّات الحَيَوِيَّة*؟

عدد الأَيَّام: ☐☐ عدد الأشهر: ☐☐

٢٠) من الَّذي وصف لك أو نصحُك بإستخدام *المُضَادَّات الحَيَوِيَّة*؟ (بإمكانك إختيار أكثر من إجابة)

○ الطّبيب ○ الأَصْدِقَاء ○ العائلة

○ الصَّيْدلانيّ ○ كانَ عندي في المنزل

٢١) في آخر مَرَّة كان عليك أن تتناول *المُضَادَّات الحَيَوِيَّة*، هل أكملت كُلّ دورة العلاج؟

○ نعم ○ كلا

٢٢) ماذا فعلت *بِالمُضَادَّات الحَيَوِيَّة* الّتي تُركت دون إستعمال؟ (بإمكانك إختيار أكثر من إجابة)

○ إحتفظت بها لكي أَستَعمِلُهَا عندما أمرض في المَرَّة القادمة

○ رميتها في الزباله أو في مياه الصَّرف الصِّحّيّ

○ أُعطيتها لأحد

○ لم يتبقى لديّ أي مُضَادَّات الحَيَوِيَّة

٢٣) في آخر مَرَّة كان عليك أن تتناول *المُضَادَّات الحَيَوِيَّة*، هل نسيت أن تأخذ أي من الجرعات؟

○ أبداً (رجاءً إنتقل إلى االسُّؤال ٢٥)

○ أحياناً (رجاءً إنتقل إلى السُّؤال ٢٤)

○ في كثيرٍ من الأحيان (رجاءً إنتقل إلى االسُّؤال ٢٤)

٢٤) ماذا فعلت عندما تخطّيت/لم تتناول جُرْعَة من *المُضَادَّات الحَيَوِيَّة*؟

○ تابعت الجُرعات التَّالِيَة بشكلٍ طبيعيٍّ

○ ضاعفت أو زدت الجُرعة الثانيَّة

○ تناولتُها عندما تذكَّرت

٢٥) في آخر مَرَّة كان عليك أن تتناول *المُضَادَّات الحَيَوِيَّة*، هل قمت بتغيير عيار الجُرعة من تِلْقَاء نفسك (بِدُون إسْتِشَارَةٍ طِبِّيَّةٍ)؟

○ أبداً (رجاءً إنتقل إلى االسُّؤال ٢٩)

○ أحياناً (رجاءً إنتقل إلى االسُّؤال ٢٦)

○ في كثيرٍ من الأحيان (رجاءً إنتقل إلى السُّؤال ٢٦)

٢٦) ماذا فعلت عندما غيَّرتُ عيار *المُضَادَّات الحَيَوِيَّة* من تِلْقَاء نفسك (بِدُون إسْتِشَارَةٍ طِبِّيَّةٍ)؟

○ تناولت منها أكثر (رجاءً إنتقل إلى االسُّؤال ٢٧ ومن ثُمَّ إلى السُّؤال ٢٩)

○ تناولت منها أقل (رجاءً إنتقل إلى االسُّؤال ٢٨)

○ أحياناً تناولت منها أكثر وأحياناً أُخرى أقل (رجاءً أجِب على السُّؤالين ٢٧ و ٢٨)

٢٧) لماذا قُمت بزيادة عيار جُرعة *المُضَادَّات الحَيَوِيَّة* من تِلْقَاء نفسك (بِدُون إسْتِشَارَةٍ طِبِّيَّةٍ)؟ (بإمكانك إختيار أكثر من إجابة)

○ نسيت أن أتناول الجُرعة السَّابقة

○ شعرت بأنّني مريضٌ جدًّا

○ لم أشعر بتحسُّن ملحوظ

○ تحسَّنت ولكن أردت أن أتحسَّن أكثر

٢٨) لماذا قُمت بتخفيض عيار جُرعة *المُضَادَّات الحَيَوِيَّة* من تِلْقَاء نفسك (بِدُون إسْتِشَارَةٍ طِبِّيَّةٍ)؟ (بإمكانك إختيار أكثر من إجابة)

○ كنتُ قَلِقًا من آثار الدواء الجانبيَّة

○ كنتُ مُتعبًا ونسيت

○ شعرت بأنّني أتناول الكثير من الدواء

○ كنتُ على عجلةٍ ونسيت

○ لا أُحِبُّ تناول الأَدْوِيَة في اللَّيل

○ شعرتُ بتحسُّن

الخَصَائِص الدِّيمُوغْرَافِيَّة العَامَّة

٢٩) حدِّد جِنسك ○ ذكر ○ أنثى

٣٠) ما هو تاريخ ميلادك؟ (يُرجى كتابة الأرقام المقابلة في الفراغات أدناه)

اليُوم	الشَّهْر	السَّنَة
□□	□□	□□□□

٣١) ما هو أعلى مستوى تعليميّ لديك؟
- ○ لم أدخل إلى المدرسة
- ○ إبتدائيّ (الصّف الأول إلى الصّف السادس)
- ○ مُتَوسِّط (الصف السَّابع إلى الصف التَّاسِع)
- ○ ثانويّ (الصف العَاشِر إلى الصف الثَّانِي عَشْر)
- ○ جامعة

٣٢) هل تعمل حاليّاً؟ ○ نعم ○ كلا

٣٣) ماذا يُغَطِّي تَأْمِينُكَ الصِّحِّيّ؟ (بإمكانك إختيار أكثر من إجابة)
- ○ ليس لديّ تأمين صِحِّيّ
- ○ التَّكلفة أو جزء من تكلفة الإستشارة الطبيَّة
- ○ التّكلفة أو جزء من تكلفة الأدويَّة

٣٤) يُرجى الإشارة إلى وضعك العائلي؟
- ○ متزوج/متزوجة (رجاءً إنتقل إلى االسُّوال رقم ٣٥)
- ○ مطلّق/مطلّقة (رجاءً إنتقل إلى االسُّوال رقم ٣٧)
- ○ أرمل/أرملة (رجاءً إنتقل إلى االسُّوال رقم ٣٧)
- ○ منفصل/منفصلة عن الزوجة/الزوج ولكن لستما مطلقين (رجاءً إنتقل إلى االسُّوال رقم ٣٥)

٣٥) ما هو أعلى مستوى تعليمي لزوجك/لزوجتك؟
- ○ لم يدخل/تدخل إلى المدرسة
- ○ إبتدائيّ (الصف الأول إلى الصف السادس)
- ○ مُتوسِّط (الصِف السَّابع إلى الصف التَّاسِع)
- ○ ثانويّ (الصف العَاشِر إلى الصف الثَّاني عَشْر)
- ○ جامعة

٣٦) هل زوجك/زوجتك يعمل/تعمل حاليّاً؟ ○ نعم ○ كلا

٣٧) ما هو عدد الأفراد الذين يعيشون في منزلك، بما في ذلك أنت؟

○ ٢ ○ ٣- ٤ ○ ٥- ٦ ○ أكثر من ٦

٣٨) هل توجد أيَّة مراكز رعاية صِحِّيَّةٌ (مثلًا مستوصفات أو مستشفيات)، موجودة بالقربِ من منطقة سكنك؟

○ نعم ○ كلا

٣٩) ما هو مجموع دخل الأسرة الشّهري (مجموع الدخل المُكتسب من جميع الأفراد الذين يعيشون في منزلك)؟
- ○ أقل من ٥٠٠ دولارٍ أمريكيٍّ في الشّهر
- ○ بين ٥٠٠ و٩٩٩ دولارٍ أمريكيٍّ في الشّهر
- ○ بين ١٠٠٠ و ١٤٩٩ دولارٍ أمريكيٍّ في الشّهر
- ○ بين ١٥٠٠ و ١٩٩٩ دولارٍ أمريكيٍّ في الشّهر
- ○ بين ٢٠٠٠ و ٢٥٠٠ دولارٍ أمريكيٍّ في الشّهر
- ○ أكثر من ٢٥٠٠ دولارٍ أمريكيٍّ في الشّهر

٤٠) هل تستشير الطَّبيب عندما تكون مريضًا؟

○ أَبَدًا ○ نَادِرًا

○ أَحْيَانًا ○ دَائِمًا (رجاءً إنتقل إلى االسُّوال رقم ٤٢)

٤١) ما هو سبب عدم إستشارتك للطَّبيب بشكلٍ دائمٍ؟ (بإمكانك إختيار أكثر من إجابة)
- ○ لا حاجة للطّبيب
- ○ الخوف من الطّبيب
- ○ عدم توفر المال الكافي
- ○ عدم توفر الوقت الكافي لزيارة الطَّبيب
- ○ فترة الإنتظار الطويلة لِيَتِمَّ فحص المريض من قبل الأطبَّاء
- ○ عدم وجود عيادة بالقرب من منزلي

٤٢) هل سبق لك أن تَلَقَّيْتَ وَ صْفَةَ طِبِّيَّةَ عبر الهاتف؟

○ نعم ○ كلا

شكرًا جزيلاً لوقتكم وتعاونكم!
